# Know Your Stripes? An Assessment of Climate Warming Stripes as a Graphical Risk Communication Format

**DOI:** 10.1111/risa.70171

**Published:** 2025-12-26

**Authors:** Ian G. J. Dawson, Danni Zhang, Shan Wang, Vanissa Wanick

**Affiliations:** ^1^ Centre for Risk Research University of Southampton Southampton UK; ^2^ Cardiff Business School Cardiff University Cardiff UK; ^3^ Winchester School of Art University of Southampton Winchester UK

**Keywords:** climate change, risk communication, risk perception, stripe graphs, warming stripes

## Abstract

Stripe graphs have emerged as a popular format for the visual communication of environmental risks. The apparent appeal of the format has been attributed to its capacity to summarize complex data in an eye‐catching way that can be understood quickly and intuitively by diverse audiences. Despite the growing use of stripe graphs among academics and organizations (e.g., Intergovernmental Panel on Climate Change [IPCC]) to communicate with both lay and expert audiences, there has been no reported empirical assessment of the format. Hence, it is not clear to what extent stripe graphs facilitate data comprehension and influence risk perceptions and the willingness to engage in mitigation actions. To address these knowledge gaps, we conducted two studies in which lay participants saw “climate warming” stripe graphs that varied in color and design. We found no evidence that traditional stripe graphs (i.e., unlabeled axes), irrespective of the stripe colors, improved the accuracy of estimates of past or predicted global temperature changes. Nor did the traditional stripe graph influence risk perceptions, affective reactions, or environmental decision‐making. Contrary to expectations, we found that viewing (cf., not viewing) a traditional stripe graph led to a lower willingness to engage in mitigation behaviors. Notably, we found that a stripe graph with date and temperature labels (cf., without labels): (i) helped participants develop more accurate estimates of past and predicted temperature changes and (ii) was rated more likable and helpful. We discuss how these and other findings can be utilized to help improve the effectiveness of stripe graphs as a risk communication format.

## 1 Introduction

**FIGURE 1:**
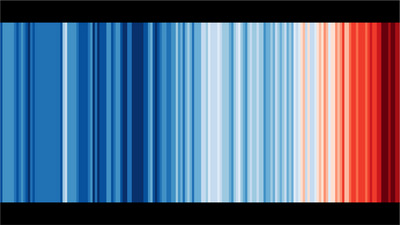
Stripe graph used in the red–blue condition of Study 1. The graph depicts changes in the average global temperature from 1850 to 2022 relative to the average temperature from 1961 to 2010. *Source*: Professor Hawkins, University of Reading.

In May 2018, Professor Ed Hawkins, University of Reading, published a graph consisting of vertical bars of equal height (i.e., stripes) in various color saturations of blue and red that represented changes in the average global annual temperature from 1850 to 2018 (e.g., see Figure 1). The graph (sometimes referred to as *warming stripes*, *climate stripes*, or *stripe graph*) was subsequently embraced by climate activists as an iconic visualization of climate change and was brought to wider public attention when posted on social media with the hashtag “#*ShowYourStripes*” (Hawkins 2019). The stripe graph has since been highlighted on multiple occasions in the mainstream media, appeared on numerous consumer goods (e.g., kitchenware, sports uniforms, and a Greta Thunberg book cover), and been projected onto natural landmarks, famous public buildings, and music festival stages. Professor Hawkins’ intention was that the graph was easy to understand and could provide a simple segue into conversations about climate change (Hawkins 2019; Irfan 2019; Rosch 2023; #ShowYourStripes 2025).

In more recent years, stripe graphs have been adopted and adapted by a variety of environmental groups and international organizations (e.g., Intergovernmental Panel on Climate Change [IPCC], World Wildlife Fund [WWF]) to communicate various environmental risks (e.g., sea‐level rises, biodiversity loss) to a range of audiences, including executives and policymakers (IPCC 2023; Richardson 2023; Skok 2022). However, although these graphs communicate information about environmental changes and appear to imply some aspect of increasing danger over time, there has been no reported assessment of the stripe graph as a format for communicating environmental and risk‐related information. Hence, it is unclear what meaning audiences extract from stripe graphs, to what extent such graphs facilitate an accurate understanding of environmental issues, and what impact the graphs might have on people's risk perceptions and behavioral intentions concerning the natural environment. Indeed, little consideration appears to have been given to the potential for the simplified design of stripe graphs to lead to misunderstandings among the target audiences. The ever‐increasing popularity, adaption, and dissemination of stripe graphs among lay individuals and experts highlights the need for (i) empirical assessments of the effectiveness of these graphs at achieving environmental risk communication goals and (ii) evidence‐based guidance for risk communicators on how stripe graphs might be better designed to fulfill more precise aims (e.g., instilling accurate knowledge of global temperature changes) in a way that matches the needs of the communicator and the skills of the target audience.

### 1.1 Climate Change, Risk Communication, Risk Perceptions, and Behaviors

In 2023, the IPCC reported that human activities, mostly via greenhouse gas emissions, have unequivocally caused climate change, with the average global temperature in the period 2011–2020 being 1.1°C above that in the period 1850–1900 (IPCC 2023). Climate change now affects weather and climate extremes in all regions of the world, with adverse impacts, losses, and damage to nature and people becoming more frequent, severe, and interconnected (IPCC 2023). The IPCC's report makes it patently clear that there is an urgent need for substantial changes in human patterns of consumption and production to achieve a sustainable future.

A fundamental starting point for tackling climate change is ensuring that individuals (i.e., laypeople and policymakers) first have accurate knowledge of climate change, the potential adverse outcomes, and the associated risks. Indeed, numerous studies show that risk perceptions have a strong positive relationship with the willingness to engage in actions that can prevent or reduce environmental problems (e.g., Dawson and Zhang 2024; Hurst Loo and Walker 2023; Stanley et al. 2021; Wynes and Nicholas 2017). For example, Leiserowitz (2005) and O'Connor et al. (1999) found that support for action on climate change was greatest among individuals with heightened risk perceptions of environmental issues. Similarly, research evidence indicates that eco‐anxiety (i.e., heightened affective states of distress associated with ecological crises; Hogg et al. 2021; Pihkala 2020) is positively related to greater engagement in pro‐environmental actions (Mathers‐Jones and Todd 2023).

Climate risk communication is one of the key processes that influences climate change knowledge and risk perceptions (Crosman et al. 2019; Pidgeon 2012; Sterman 2008). In recent years, visual and graphical formats (e.g., bar charts, line graphs, maps, and artistic representations) have been championed as an effective medium for the general improvement of risk communications (e.g., Meyer et al. 1997; Okan et al. 2018; Smerecnik et al. 2010; Stone et al. 2017), and more specifically, for improving the efficacy of communications specifically about climate change (Bruine de Bruin et al. 2024; Kaye et al. 2012; Newell et al. 2016; O'Neill and Smith 2014). Graphical communication formats are reported to have many benefits, which include the potential for (i) attracting and holding attention more effectively, (ii) reducing cognitive effort for information processing, (iii) presenting content that is more memorable than text/verbatim formats, (iv) transcending linguistic, educational, and cultural barriers, (v) depicting alternative scenarios and outcomes, and (vi) increasing the personal relevance and emotional salience of the issue and potential outcomes (Hahn and Berkers 2021; Kaye et al. 2012; Li et al. 2023; Newell et al. 2016; O'Neill and Smith 2014).

### 1.2 Stripe Graphs as a Format for Graphical Climate Change Communication

Stripe graphs are a contemporary and pertinent example of graphical climate change communication (e.g., Figure 1). This format was first introduced in 2018 and has subsequently become extremely popular among lay audiences and, notably, among experts as a method for communicating environmental data and issues (Dixon 2023; Lasagna et al. 2024; Skok 2022). For example, the IPCC used a complex stripe graph in the 2023 Sixth Assessment Report, which depicted, among other things, different future scenarios, a color‐temperature guide, and details of climate change impacts for people of different ages. Similarly, Professor Miles Richardson at the University of Derby created a stripe graph in color saturations of green, yellow, and gray depicting the global loss of biodiversity from 1970 to 2020 (#BiodiversityStripes 2025).

It is important to note the ways that the design of Hawkins’ original “warming stripes” graph varies from a traditional bar graph. Hawkins’ graph uses bars that (i) are of equal height, (ii) directly touch one another, and (iii) are in two colors (blue and red) of varying saturations. More specifically, variation in color and saturation, rather than bar height, is the key mechanism for communicating information about variations in temperature, with darker saturations of blue (red) corresponding to a relatively lower (higher) annual average global temperature. Past research findings support that, typically, audiences intuitively understand that blue represents colder temperatures and red represents hotter temperatures (Braun et al. 1995; Schneider and Nocke 2018; Tang and Rundblad 2015). Thus, the semantics of the color scheme in Hawkins’ stripe graph would potentially be highly familiar to the target audience. However, it might also convey information about risk as well as temperature. Research evidence suggests that the color red (blue) is often perceived to represent the highest (lowest) level of danger or maximal (minimal) hazardous conditions when featured in graphical communications (Braun et al. 1995; Dunlap 1986; Rodriguez 1991). Furthermore, evidence indicates that red can function as an implicit “stop signal” that operates outside of conscious awareness (Genschow et al. 2012). Consequently, the colors in Hawkins’ stripe graph could be interpreted as (ambiguously) communicating information about changes in risk/danger as well as temperature, and it is not clear how these two different interpretations might influence the audience's knowledge and behaviors.

Another key design feature of Hawkins’ original graph is the absence of labels on the graph's axes. This is a deliberate design feature to make the graph accessible to audiences with diverse levels of scientific literacy and to prevent the graph from appearing esoteric (#ShowYourStripes 2025). However, the absence of text labels on the graph might have undesirable effects. For example, O'Neill and Smith (2014) argue that the absence of syntax in graphical climate change communications can mean that the conveyed message lacks precision and is open to misinterpretation (also see Grøndahl et al. 2011). For a stripe graph, such misinterpretations might relate to the time period depicted, the region the data were collected from, the metrics reported (e.g., Celsius vs. Fahrenheit), or the magnitude of the temperature increases. Furthermore, several studies show that the ability to understand graphically presented information (known as “graph literacy”) varies significantly between individuals, and that graph literacy can affect perceptions of risk and benefits, therefore influencing decisions and behaviors (Galesic and Garcia‐Retamero 2011; Okan et al. 2012, 2019).

As highlighted above, it is possible that stripe graphs may be interpreted in a variety of ways and that this may result in different evaluations and influences of the communicated data. Some of these interpretations may involve misunderstandings that might be attributable to the format's simplified design. This may have implications for the extent to which individuals have accurate knowledge of climate change and/or are motivated to engage in mitigation actions. For example, if someone who views a climate stripes graph infers that there has been a 7°C increase in the average annual global temperature between 1850 and 2023, they may perceive it to be a positive situation if climate scientists report a further increase of 2.5°C is forecast in the average annual global temperature by 2050 (because this future increase would seem small relative to the perceived historical increase). Such misinterpretations of predicted climatic changes could result in large underestimations of risk, which could attenuate the extent to which the person engages in climate mitigation actions.

### 1.3 The Present Studies

Considering the growing adaptation and dissemination of stripe graphs that report data on critical environmental issues, the need for empirical assessments of these communication formats is increasingly important. It is essential to understand the audience's interpretation of stripe graphs and assess the extent to which the graphs influence knowledge, perceptions, and the willingness to engage in mitigation behaviors. Such assessments can facilitate the production of comprehensive, evidence‐based guidance on how the content of the graphs can be enhanced to improve the format's capacity to effectively communicate environmental risks to target audiences.

To initiate this important line of research, we conducted two empirical studies. Study 1 examined the extent to which knowledge, perceptions, and behavioral intentions concerning climate change were influenced by viewing (cf., not viewing) a stripe graph and by the colors of the stripes. Although the study was predominantly exploratory, we anticipated that a stripe graph in blue–red saturations might elicit the greatest increases in risk perceptions and behavioral intentions because red typically indicates a high level of danger in graphical communications. Study 2 examined the extent to which knowledge, perceptions, and behavioral intentions might be influenced by the addition of data labels (i.e., time and temperature) on the axes of a blue–red stripe graph. Again, the study was exploratory in nature, but we anticipated that the inclusion of labels would improve knowledge accuracy concerning average global temperature changes.

## 2 Method

### 2.1 Study 1

Our first study (OSF preregistration: https://doi.org/10.17605/OSF.IO/PQEAB) assessed whether climate change risk perceptions, knowledge, and behavioral intentions differed among individuals who viewed a stripe graph featuring blue and red stripes compared to individuals who (i) viewed a stripe graph featuring two alternatively colored stripes or (ii) did not view a stripe graph.

#### 2.1.1 Participants

Power analysis using G*Power indicated a sample size of ≥305 was needed to detect a medium effect size of 0.25 at the standard 0.05 alpha error probability when using a one‐way ANOVA. We recruited 315 adult participants using the academic research platform Prolific (www.prolific.com) and paid each participant £1.50. We specified that all participants must be UK residents and have (i) English as a first language, (ii) completed at least 20 previous studies on Prolific, (iii) a Prolific approval rating of ≥95%, and (iv) no visual impairments. None of the participants failed our Prolific‐compliant “instructional manipulation check” (IMC; Oppenheimer et al. 2009). The mean age of the sample was 39.6 years (SD = 13.8), and 179 (56.8%) had obtained an undergraduate degree or higher qualification: 157 identified as male, 157 as female, and one as “other (e.g., non‐binary).”

#### 2.1.2 Materials and Procedure

**FIGURE 2:**
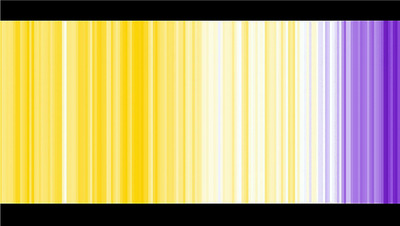
Stripe graph used in the yellow–purple condition of Study 1. The graph depicts changes in the average global temperature from 1850 to 2022 relative to the average temperature from 1961 to 2010. *Source*: Figure created by authors.

Using a between‐subjects design, we randomly allocated our participants into one of three conditions and asked them to complete a purpose‐made questionnaire hosted in Qualtrics. Participants in the “control condition” (*n* = 104) were not presented with a stripe graph while answering items concerning key variables. Participants in the “blue–red condition” (*n* = 105) were first presented with a “traditional” climate stripes graph that featured conjoined vertical bars of equal height in various saturations of blue and red. The saturations represented changes in the average global temperature from 1850 to 2022 relative to the period 1961–2010 (see Figure 1). Participants in the “yellow–purple condition” (*n* = 106) were first presented with a climate stripes graph that featured conjoined vertical bars of equal height in various saturations of yellow and purple representing changes in the average global temperature (see Figure 2). The yellow–purple stripe graph was constructed in Microsoft Excel using temperature observation data collaboratively compiled by the Met Office Hadley Centre and the Climatic Research Unit at the University of East Anglia (Met Office 2025). This graph also depicted the average global temperature from 1850 to 2022 relative to the period 1961–2010. Hence, the graph replicated the same pattern of saturation variation shown to participants in the blue–red stripe graph condition. We elected to use yellow and purple because these colors (i) provided a distinct alternative to those used in the blue–red condition, (ii) avoided some colors with obvious connections to traditional semantic messages (e.g., green = positive), and (iii) provided sufficient contrast with each other to enable audiences to make comparisons between variations in color saturation (e.g., we found that grayscale saturations did not provide a visual distinction between an extreme cold temperature and an extreme hot temperature) (Braun et al. 1994; Schneider and Nocke 2018). Neither the red–blue graph nor the yellow–purple graph featured a heading/title or labels on the axes, thus replicating the style and format most used within climate stripe graphs displayed in public contexts. Participants in both the blue–red and yellow–purple conditions were first instructed to “… *take a moment to study this graphical image, which represents how the Earth's temperature has been changing*” (we configured Qualtrics to prevent participants from skipping past the image for at least 20 s). To enable us to assess the direct psychological influence of the graphs, we made the graphs visible to the participants in these two conditions while they answered specific items in the questionnaire (designated in the following paragraph with an “*”).

To measure the participant's knowledge of past temperature changes and their estimates of future temperature changes, we asked two questions: “*By how many degrees Celsius do you think the average annual global temperature has changed since the year 1850?*”* and “*By how many degrees Celsius do you think the average annual global temperature will increase between now and the year 2050?*”* Participants could respond to each question using a sliding response scale ranging from −20°C to 20°C. To measure the perceived risk of climate change, we asked all participants to use an 11‐point scale (0 = strongly disagree, 10 = strongly agree) to express their agreement that (i) “*humanity is not doing enough to tackle climate change*”*, (ii) “*the average global annual temperature will rise to dangerous levels during this century*”*, (iii) “*climate change will pose a threat to the existence of humanity during this century*”*, and (iv) “*the individual actions I can take to tackle climate change are too small to make a difference to global annual temperatures*”*. Participants in the blue–red and yellow–purple conditions used an 11‐point scale to evaluate the stripe graph on the following criteria: (i) like the way information is presented [0 = do not like it at all, 10 = like it a lot], (ii) trust [0 = not trust at all, 10 = complete trust], (iii) provides accurate information [0 = not at all accurate, 10 = extremely accurate], and (iv) helpful for understanding global temperature changes [0 = not at all helpful, 10 = extremely helpful] (Okan et al. 2018)*.

To measure affective responses to climate change, all participants used 11‐point scales (0 = not at all, 10 = a great deal) to express the extent to which thinking about global annual temperature changes brought up positive affective states of “*happiness*,” “*hope*,” “*inspiration*,” “*awe*,” and “*curiosity*,” and negative affective states of “*guilt*,” “*sadness*,” “*fear*,” “*anger*,” and “*uneasiness*” (Li et al., 2023)*.

To measure the participants behavioral intentions concerning climate change mitigation, we asked them to use an 11‐point scale (0 = not at all, 10 = extremely willing) to indicate their willingness to (i) reduce their annual air travel by 20%, (ii) reduce their annual road travel in a personal vehicle by 20%, (iii) reduce their annual household energy consumption by 20%, (iv) reduce their annual consumption of consumer goods and services by 20%, (v) vote for a political party/candidate committed to reducing or stopping global warming, (vi) vote for a political party/candidate committed to increasing taxes on fossil fuels, and (vii) talk about climate change with others who do not agree with their view on this topic (Hurst Loo and Walker 2023)*. Moreover, to measure potential variations in decision‐making concerning climate change, we asked all participants to imagine that they were responsible for deciding how the UK Government should allocate funds for dealing with the issues of (i) public health, (ii) environment and climate change, and (iii) crime and policing. We asked them to indicate the percentage of funds (totaling 100%) that they would allocate to each of the three issues.

We asked our participants in the blue–red and yellow–purple conditions “*to state how many times they had seen a climate stripes graph (even if the stripes were a different color to those shown in this study) before you participated in this study*.” At the end of the procedure, participants in the control condition were shown the stripe graph from the blue–red condition and asked to indicate how many occasions they had seen a similar image before.

Finally, our participants completed the short graph literacy scale (SGLS: Okan et al. 2019). The SGLS consisted of four questions that measure the ability of individuals to understand graphical data, extract information from graphs, and make inferences beyond the depicted data in graphs. For exploratory purposes, we also asked our participants to complete a nine‐item scale measuring worldviews (Smith and Leiserowitz 2014) and a 13‐item scale measuring eco‐anxiety (Hogg et al. 2021) because both of these constructs have been found to have strong associations with risk perceptions and behaviors concerning environmental issues (Leiserowitz 2005; Mathers‐Jones and Todd 2023; O'Connor et al. 1999; Pihkala 2020).

#### 2.1.3 Data Preparation and Analysis

Scales were formed for single constructs (e.g., risk perception and eco‐anxiety) from the respective response data and assessed for reliability using Cronbach's *α*. ANOVAs and a multiple regression were then performed to assess the relationships between the conditions and the other key variables.

#### 2.1.4 Analysis and Results

According to the IPCC, the average global temperature in the period 2011–2020 was 1.1°C above that in the period 1850–1900 (IPCC 2023). Our sample's mean judgment of the average global temperature change since 1850 was 6.79°C (SD = 4.97). A one‐way ANOVA identified no significant difference in these judgments between the three conditions, *F*(2, 312) = 2.28, *p* = 0.104 (see Table 1). We categorized our participants’ judgments of the average global temperature change as “accurate” if it was either 0°C, 1°C, or 2°C (i.e., we used an approximate tolerance of −/+1 either side of 1.1°C) and categorized all other judgments as “inaccurate.” A 3 × 2 cross‐tabulated Pearson chi‐square test (condition × judgment accuracy) identified no significant difference in the proportion of accurate judgments (*n* = 54; 17.1% of sample) across the three conditions, *χ* ^2^ = 1.61, df = 2, *p* = 0.446. However, a Pearson correlation test identified a strong significant and negative relationship between our sample's graph literacy scores (*M* = 2.70, SD = 0.98) and their judgments of the past temperature change, *r* = −0.21, *N* = 315, *p* < 0.001.

**TABLE 1:** Study 1: Participants’ mean responses to questions concerning (i) past and future changes in annual average global temperature, (ii) risk perceptions of climate change, (iii) willingness to adopt mitigation behaviors, (iv) stripe graph evaluations, (v) affective reactions to climate change, and (vi) percentage of funds allocated to societal issues (*N* = 315).

Item	Control condition mean (SD)	Red–blue condition mean (SD)	Yellow–purple condition mean (SD)	Total mean (SD)
***Average annual global temperature changes***				
Average global temperature change since 1850	6.43°C (4.69)	7.63°C (4.71)	6.31°C (5.40)	6.79°C (4.97)
Average global temperature change between the present time and 2050	5.69°C (5.02)	7.09°C (5.92)	5.84°C (5.71)	6.21°C (5.58)
***Risk perceptions***				
*(Response scale: 0 = strongly disagree, 10 = strongly agree)*				
Humanity is not doing enough to tackle climate change	7.64 (2.49)	7.76 (2.08)	7.71 (2.38)	7.70 (2.32)
The average global temperature will rise to dangerous levels during this century	7.35 (2.53)	7.62 (2.40)	7.63 (2.36)	7.53 (2.43)
Climate change poses a threat to the existence of humanity during this century	6.75 (2.80)	6.83 (2.81)	6.97 (2.78)	6.85 (2.79)
The individual actions I can take to tackle climate change are too small to make a difference to global annual temperatures	7.67 (2.48)	7.29 (2.43)	7.24 (2.85)	7.40 (2.60)
Overall perceived risk scale				7.37 (2.15)
***Willingness to employ mitigation behaviors***				
*(Response scale: 0 = not at all, 10 = extremely willing)*				
Reduce annual air travel by 20%	6.35 (3.39)	5.37 (3.43)	5.24 (3.43)	5.65 (3.44)
Reduce annual road travel in personal vehicle by 20%	5.94 (3.20)	5.64 (3.10)	4.83 (3.12)	5.47 (3.17)
Reduce annual household energy consumption by 20%	6.08 (2.82)	6.45 (2.66)	5.10 (2.91)	5.87 (2.85)
Reduce annual consumption of consumer goods and services by 20%	6.09 (2.61)	6.03 (2.90)	5.08 (3.01)	5.73 (2.88)
Vote for political party/candidate committed to reducing or stopping global warming	6.80 (3.16)	6.42 (3.15)	6.36 (3.22)	6.52 (3.17)
Vote for a political party/candidate committed to increasing taxes on fossil fuels	6.13 (3.56)	6.03 (3.34)	5.86 (3.27)	6.00 (3.38)
Talk about climate change with others who do not agree with my view on this topic	6.24 (2.79)	5.48 (2.92)	5.47 (3.15)	5.73 (2.97)
Overall willingness to mitigate scale				5.85 (2.48)
***Stripe graph evaluations***				
Like the way the information in the graph is presented *(Response scale: 0 = do not like it at all, 10 = like it a lot)*	—	5.89 (2.84)	3.41 (2.88)	4.64 (3.11)
Trust the information in the graph *(Response scale: 0 = not trust at all, 10 = complete trust)*	—	5.70 (2.44)	4.42 (2.65)	5.06 (2.62)
Think the graph provides accurate information *(Response scale: 0 = not at all accurate, 10 = extremely accurate)*	—	5.48 (2.39)	4.18 (2.67)	4.82 (2.61)
Graph is helpful for understanding global temperature changes *(Response scale: 0 = not at all helpful, 10 = extremely helpful)*	—	6.30 (2.74)	3.25 (2.83)	4.77 (3.17)
Overall graph evaluation scale				4.82 (2.56)
***Affective reactions to global temperature changes***				
*(Response scale: 0 = not at all, 10 = a great deal)*				
Happiness	1.52 (1.85)	1.28 (1.49)	1.35 (1.62)	1.38 (1.66)
Hope	2.36 (2.24)	2.55 (2.04)	2.91 (2.32)	2.61 (2.21)
Inspiration	2.13 (1.98)	2.50 (2.20)	2.42 (1.96)	2.35 (2.06)
Awe	2.74 (2.39)	3.19 (2.42)	2.82 (2.67)	2.92 (2.50)
Curiosity	4.79 (2.35)	4.61 (2.51)	4.75 (2.53)	4.72 (2.46)
Overall positive affect scale				2.84 (1.54)
Guilt	5.21 (2.80)	5.11 (2.85)	5.21 (2.90)	5.18 (2.84)
Sadness	6.63 (2.47)	6.69 (2.60)	6.76 (2.68)	6.70 (2.58)
Fear	6.46 (2.58)	6.32 (2.68)	6.56 (2.72)	6.45 (2.66)
Anger	5.93 (2.49)	5.75 (2.90)	6.16 (2.79)	5.95 (2.73)
Uneasiness	6.73 (2.48)	6.99 (2.49)	6.75 (2.57)	6.82 (2.51)
Overall negative affect scale				6.23 (2.44)
***Percentage of funds allocated to societal issues***				
*(Response scale: 0%–100%)*				
Public health	42.86 (10.44)	41.31 (11.33)	43.73 (11.02)	42.64 (10.95)
Environment and climate change	28.05 (12.59)	27.26 (11.81)	28.91 (12.39)	28.08 (12.25)
Crime and policing	29.09 (10.18)	31.43 (11.04)	27.35 (9.16)	29.29 (10.26)

*Note*: Data are displayed separately for the control condition (*n* = 104), blue–red graph condition (*n* = 105), and yellow–purple condition (*n* = 106).

The IPCC (2023) currently reports the future scenario of a “*very high greenhouse gas emissions scenario*” (i.e., current “worst case” scenario) as resulting in a ∼2.5°C increase by 2050 in the average global temperature above the pre‐industrial period of 1850–1900. Our sample's mean estimate of the average global temperature change between the present time and 2050 was 6.21°C (SD = 5.58). A one‐way ANOVA identified no significant difference in these “future temperature” estimates between the three conditions, *F*(2, 312) = 1.99, *p* = 0.139 (see Table 1). A Pearson correlation test identified a strong significant and negative relationship between our sample's graph literacy scores and their estimates of the future temperature change, *r* = −0.28, *N* = 315, *p* < 0.001.

We also performed two independent samples *t*‐tests to help determine what impact viewing a climate stripes graph for the first time could have on judgments of past and future temperature changes. For this analysis, we first selected only those participants in our sample who reported not having seen a stripe graph before the study (*n* = 211). From this sub‐sample, we ran *t*‐tests comparing the temperature estimates of the participants in the control group (*n* = 88) with participants in the stripe graph conditions (*n* = 123). The first test identified that judgments of past temperature changes were significantly higher (*t*(209) = 2.39, *p* < 0.01) among participants who saw a stripe graph for the first time during the study (*M* = 7.47, SD = 5.0, *n* = 123) compared to participants in the control group, that is, those who had not seen a stripe graph either before or during the study (*M* = 5.88, SD = 4.5, *n* = 88). The second test identified that judgments of future temperature changes were also significantly higher (*t*(209) = 2.12, *p* < 0.05) among participants who saw a stripe graph for the first time during the study (*M* = 6.99, SD = 5.9, *n* = 123) compared to participants in the control group (*M* = 5.36, SD = 4.9, *n* = 88).

Table 1 shows the mean responses to the main measures in our questionnaire. We created separate scales from the items used to measure, respectively, perceived risk (Cronbach's *α* = 0.87), graph evaluations (Cronbach's *α* = 0.91), positive affective (Cronbach's *α* = 0.73), negative affect (Cronbach's *α* = 0.94), and willingness to mitigate (Cronbach's *α* = 0.90). We then performed separate one‐way ANOVAs to assess for between‐conditions differences in overall perceived risk, overall positive affect, overall negative affect, and overall willingness to mitigate. The results identified no significant difference between the conditions for all constructs, *F*s(2, 312) ≤ 2.89, *p*s ≥ 0.057. We then performed an independent samples *t*‐test to determine whether the overall graph evaluations varied between the blue–red condition and the yellow–purple condition. The test identified that the overall evaluation in the blue–red condition (*M* = 5.84, SD = 2.28) was significantly higher than in the yellow–purple condition (*M* = 3.81, SD = 2.43), *t*(209) = 6.26, *p* < 0.001. We performed follow‐up *t*‐tests on each of the four items in the overall evaluation scale and found that likeability, trustworthiness, information accuracy, and helpfulness were all significantly higher (with Bonferroni correction of *p* = 0.05/4 = 0.0125) in the blue–red condition than in the yellow–purple condition, *t*s(209) ≥ 3.68, *p*s < 0.001.

We also performed a further ANOVA to assess for between‐condition differences in the proportion of funds that our participants believed the UK Government should allocate to environment and climate change (see Table 1). The result showed no difference in the mean allocation in the control condition (*M* = 28.05%, SD = 12.59), the blue–red graph condition (*M* = 27.26%, SD = 11.81), and the yellow–purple condition (*M* = 28.91%, SD = 12.39), *F*(2, 312) = 0.48, *p* = 0.620.

Considering the importance of public engagement in climate change mitigation behaviors, we conducted an exploratory multiple regression analysis to determine which factors might be associated with such behaviors. We performed a forced entry linear regression with willingness to mitigate as the outcome variable and overall perceived risk, condition (0 = control condition, 1 = graph seen conditions), past temperature change, future temperature change, positive affect, negative affect, graph literacy, worldviews, eco‐anxiety, and prior experience of stripe graphs as the predictors. Tables 2 and 3 show the correlations and coefficients, respectively. The analysis revealed that perceived risk, condition, negative affect, worldviews, and eco‐anxiety were significant predictors, with the regression model explaining 50% of the variance in willingness to mitigate.^1^ To explore the finding that the condition was a predictor, we performed an independent samples *t*‐test, which showed that willingness to mitigate was significantly greater among participants in the control condition (*M* = 6.231, SD = 2.292) compared to participants in the stripe graph conditions (*M* = 5.667, SD = 2.550), *t*(313) = 1.91, *p* = 0.029.

**TABLE 2:** Study 1: Correlations between assessed variables (*N* = 315).

	1	2	3	4	5	6	7	8	9	10	11
1. Overall mitigation willingness	1										
2. Overall perceived risk	0.616^***^	1									
3. Condition	−0.107^*^	0.006	1								
4. Past temperature	0.048	0.017	0.051	1							
5. Future temperature	0.048	0.127^*^	0.065	0.636^***^	1						
6. Positive affect	0.002	−0.079	0.041	−0.043	0.067	1					
7. Negative affect	0.639^***^	0.749^***^	0.007	0.039	0.096^*^	0.062	1				
8. Graph literacy	0.045	0.108^*^	−0.074	−0.206^***^	−0.278^***^	−0.081	0.065	1			
9. Worldviews	0.463^***^	0.516^***^	−0.073	−0.025	0.003	−0.146^**^	0.442^***^	0.130^*^	1		
10. Eco‐anxiety	0.306^***^	0.264^***^	−0.026	0.067	0.111^*^	0.065	0.347^***^	−0.149^**^	0.108^*^	1	
11. Stripe graph past experience	0.026	0.043	0.095^*^	−0.008	−0.013	0.055	0.027	0.052	0.019	0.059	1

* *p* < 0.05.

** *p* < 0.01.

*** *p* < 0.001.

**TABLE 3:** Study 1: Regression of assessed variables on overall willingness to engage in climate change mitigation behaviors (*N* = 315).

	Unstandardized coefficients		Standardized coefficients
	*b*	SE	*β*
Overall perceived risk	0.307	0.077	0.266^***^
Condition	−0.519	0.217	−0.099^*^
Past temperature	0.042	0.027	0.084
Future temperature	−0.038	0.025	−0.085
Positive affect	0.052	0.069	0.032
Negative affect	0.351	0.068	0.336^***^
Graph literacy	−0.061	0.111	−0.024
Worldviews	0.258	0.074	0.170^***^
Eco‐anxiety	0.474	0.218	0.096^*^
Stripe graph past experience	0.005	0.040	0.005
*R^2^*			0.496^***^
*F*(10,304)			29.944

*Note*: Variance inflation factor (VIF) and tolerance statistics showed no evidence of multicollinearity.

* *p* < 0.05.

** *p* < 0.01.

*** *p* < 0.001.

#### 2.1.5 Discussion

Our samples’ judgments of past and future annual average global temperatures were much higher than both the historical data and the IPCC's future “worst‐case scenario.” Notably, Study 1 found no evidence to suggest that the stripe graphs, irrespective of the colors used in the graph, improved the accuracy of the audience's knowledge of past or predicted temperature changes. A further interesting finding was that viewing a stripe graph for the first time was associated with significantly higher (i.e., less accurate) estimates of past and future global temperature changes. This suggests that when first viewing a climate stripes graph, individuals may interpret the dramatic changes in the stripe colors and saturations as an indication that their extant estimates of past and future global temperatures are too low and, thus, need to be revised upwards. Although one of the objectives of the climate stripe graphs may be to alert people to the past magnitude of (and future potential for) global temperature increases (#ShowYourStripes 2025), the overall results from Study 1 indicate that heightened temperature estimates among stripe graph audiences may only be a temporary effect particular to first viewings. Furthermore, it can be argued that if viewing a climate stripes graph increases the extent to which the audience's knowledge of temperature changes is inaccurate (as was the case for the “first time viewers” in Study 1), this may be counterproductive in helping people to better understand and address climate change. A particularly interesting finding from Study 1 was that, compared to the yellow–purple graph, the blue–red graph was considered much more likeable, trustworthy, accurate, and helpful. Given that both graphs reported the same data in the same saturation pattern, this finding indicates that the color schemes were responsible for these between‐condition variations in the subjective evaluations of the graphs. The participants’ preference for the blue–red graph might be because the colors correspond with those typically used in the UK to represent, respectively, cooler and warmer temperatures (Tang and Rundblad 2015) and, therefore, the participants considered the intended meaning of the graphical data to be more intuitively obvious. However, it is worth considering that the colors used in the graphs have no relevance to the accuracy and trustworthiness of the data that are depicted. Hence, our results suggest that the colors used in stripe graphs can have unintended and illogical effects on the audiences’ subjective evaluations of the data that the graphs depict.

In addition, Study 1 identified that the stripe graphs did not influence perceived risk, affect (i.e., our quantitative measures of positive and negative affect), or environmental decision‐making (i.e., our item concerning the allocation of government funds to address environmental issues and climate change). However, our regression analysis did find that participants who saw the stripe graphs (cf., those who did not) generally had a *lower* willingness to engage in mitigation behaviors. It is surprising that the stripe graphs had this attenuating effect, and the underlying reason for this is not clear, particularly because it appears to have occurred independently of any variations in our participants’ risk perceptions. One potential explanation for this attenuation is that the graphs may have elicited or enforced a sense of helplessness or apathy among the audience, which ultimately depressed the perceived efficacy of personal mitigative actions (Gunderson 2023). Another explanation could be that the heavy presence of red in the graph was implicitly interpreted as a “stop signal” that attenuated the motivation to engage in mitigation behaviors (Genschow et al. 2012). Alternatively, the graphs may have focused the participants’ attention on the historic nature of climate change rather than on the merits of mitigation actions in the present or future (Doyle 2009).

Our regression analysis also found that the participants’ willingness to engage in mitigation behaviors was strongly associated with perceived risk, negative affect, eco‐anxiety, and worldviews. This is consistent with several other studies that show heightened risk perceptions, negative affect, and eco‐anxiety are related to a greater desire to engage in mitigative actions for environmental issues (e.g., Dawson and Zhang 2024; Leiserowitz 2005, 2006; Mathers‐Jones and Todd 2023). Likewise, as shown in our study, previous studies have found that individuals with more egalitarian‐communitarian worldviews (who typically advocate for more equal distribution of wealth, resources, risk, and responsibilities, and for participatory approaches to societal issues) are typically more motivated to engage in and support pro‐environmental actions than individuals with individualistic‐hierarchical worldviews (who typically advocate for more individual autonomy within a hierarchical system in which specific groups may hold more power and responsibility for managing societal issues; Hornsey 2021).

Finally, although we found that the stripe graphs used in Study 1 did not influence the accuracy of judgments of past and future temperature changes, the results did indicate that there was a positive relationship between graph literacy and the accuracy of such judgments. This suggests that, irrespective of exposure to climate stripe graphs, individuals with higher graph literacy may be better able to interpret, evaluate, and/or recall data on annual global temperature changes.

### 2.2 Study 2

Study 1 identified that the stripe graphs did not affect whether participants formed accurate knowledge of past and projected annual average global temperature changes. An obvious explanation for this would be that the stripe graphs in Study 1 did not explicitly feature any numerical data on global temperature changes over time, so the participants received no information that would prompt a revision of their existing knowledge. This implies that the provision of such numerical data on a stripe graph could lead to improvements in knowledge accuracy. To assess this proposition, we conducted a second study (OSF preregistration: https://doi.org/10.17605/OSF.IO/BC4T8) that examined the relative influence of three stripe graph designs as featured on Professor Hawkins’ “*ShowYourStripes*” website (https://showyourstripes.info). Specifically, Study 2 assessed the relative influence of “climate warming” stripe graphs that featured (i) no labels, (ii) date labels only, and (iii) date and temperature labels. As in Study 1, we examined the extent to which the different graphs facilitated data comprehension and influenced risk perceptions and behavioral intentions. Data gathering for Study 1 was completed in August 2024. The data were analyzed prior to gathering data for Study 2, which was completed in October 2024.

#### 2.2.1 Participants

Consistent with the same power analysis requirements for Study 1, we recruited from Prolific a sample of 317. Participants from Study 1 were prohibited from participating in Study 2. All participants met the same criteria used in Study 1 and were each paid £1.50. Two participants failed the IMC, leaving a final sample of 315 participants. The mean age of the sample was 42.9 years (SD = 13.9), and 180 (57.1%) had obtained an undergraduate degree or higher qualification. 158 identified as male, 155 as female, one as “other (e.g., non‐binary),” and one declined to state their gender.

#### 2.2.2 Materials and Procedure

**FIGURE 3:**
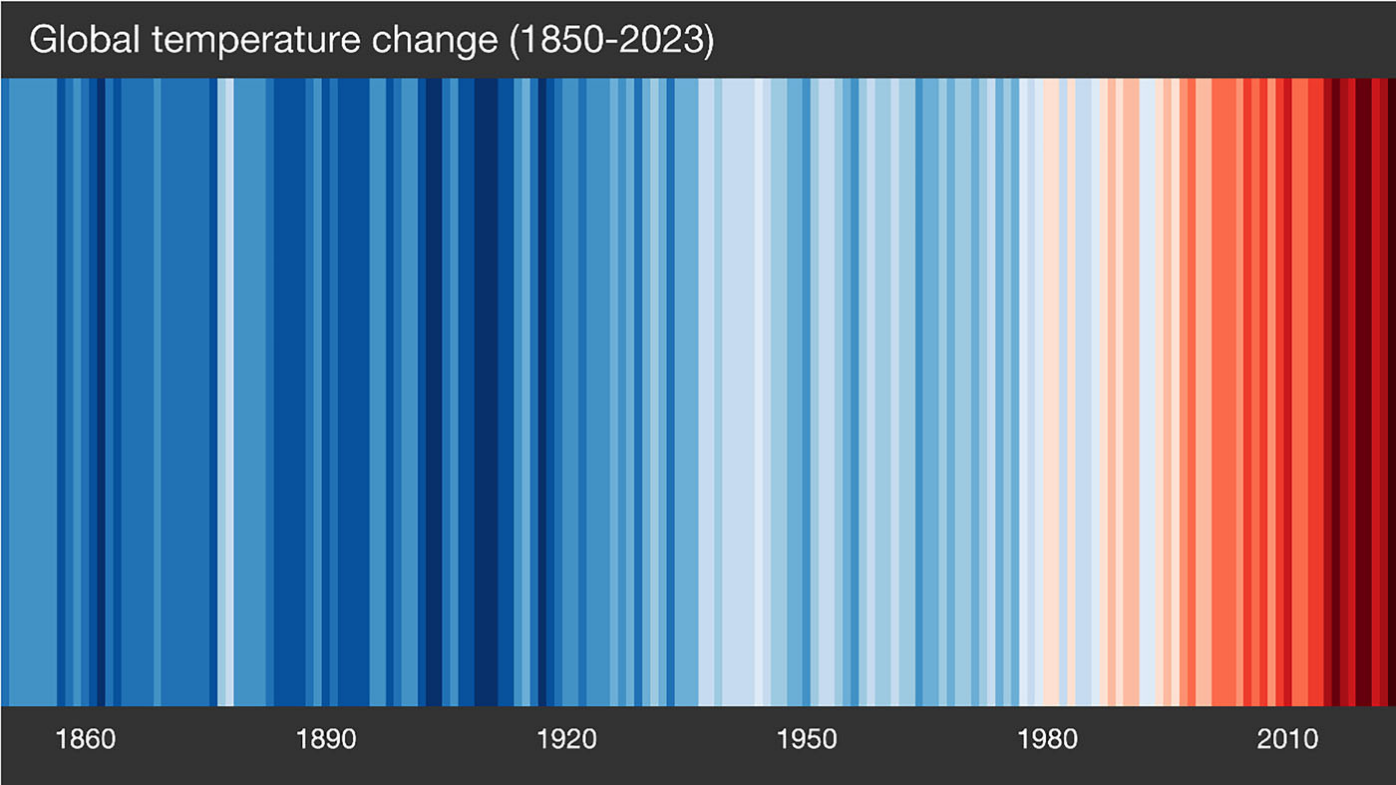
Stripe graph used in the dates‐only condition of Study 2. The graph depicts changes in the average global temperature from 1850 to 2023 relative to the average temperature from 1961 to 2010. The graph features date labels on the *x*‐axis. *Source*: Professor Hawkins, University of Reading.

**FIGURE 4:**
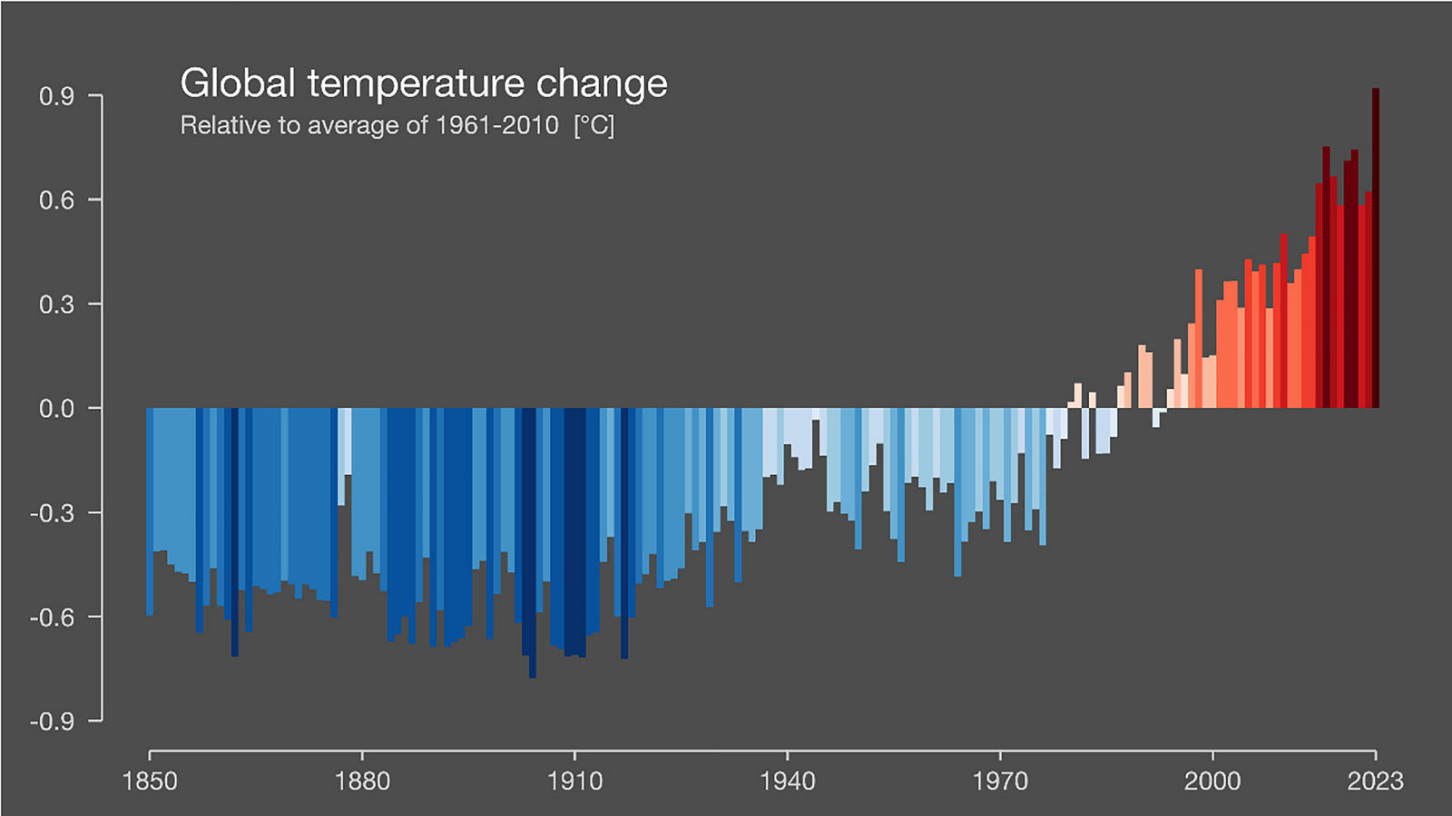
Stripe graph used in the dates and temperature condition of Study 2. The graph depicts changes in the average global temperature from 1850 to 2023 relative to the average temperature from 1961 to 2010. Graph features dates on the *x*‐axis and temperatures on the *y*‐axis. *Source*: Professor Hawkins, University of Reading.

Following a between‐subjects design, participants were randomly allocated into one of three conditions and then completed a questionnaire hosted in Qualtrics. Participants in the “no labels condition” (*n* = 109) were presented with the blue–red stripe graph shown in Figure 1. Participants in the “dates only condition” (*n* = 105) were presented with a blue–red stripe graph like that used in the no labels condition, except this graph also had the short title “*Global temperature change (1850–2023)*” displayed at the top, and year labels for every 30 years during the period 1850–2010 displayed on the *x*‐axis (see Figure 3). Participants in the “dates and temperatures condition” (*n* = 101) were presented with a blue–red stripe graph that featured (i) the title “*Global temperature change—Relative to average of 1961–2010 [°C]*” displayed at the top, (ii) an *x*‐axis with year labels for every 30 years during the period 1850–2010, plus a year label for 2023, (iii) a *y*‐axis with the temperature in degrees Celsius ranging from −0.9 to 0.9, and (iv) stripes/bars of varying heights in saturations of blue or red; the stripes displayed below 0°C on the *y*‐axis were in blue saturations, and the stripes displayed above 0°C were in red saturations (see Figure 4). We elected to use these three different versions of Professor Hawkins’ stripe graph because they are freely available in the public domain (regularly updated versions can be accessed at: https://showyourstripes.info).

Participants in all conditions were first asked to “… *take a moment to study this graphical image, which represents how the Earth's temperature has been changing*” (again, the image was displayed for 20 s before the option to progress became available). The participants then answered the same following items used in Study 1 (“*” denotes that the condition‐relevant graph was visible to the participants while answering the items): (i) average annual global temperature change since 1850*, (ii) average annual global temperature increase by 2050*, (iii) perceived risk* [four items], (iv) affective responses to climate change* [10 items], (v) graph evaluations* [four items], (vi) willingness to adopt mitigation actions* [seven items], (vii) allocation of government funds to environment and climate change, (viii) the SGLS [four items], (ix) eco‐anxiety [13 items], (x) worldviews [nine items], and (xi) number of previous exposures to stripe graphs.

#### 2.2.3 Data Preparation and Analysis

The analysis for Study 2 followed the same approach as used in Study 1.

#### 2.2.4 Analysis and Results

The sample's mean judgment of the average global temperature change since 1850 was 6.14°C (SD = 5.17). A one‐way ANOVA identified a significant difference in these judgments between the three conditions, *F*(2, 312) = 5.99, *p* = 0.003 (see Table 4). A planned contrast showed that temperature judgments in the dates and temperatures condition (*M* = 4.86°C, SD = 4.76) were significantly lower than judgments in the no labels condition (*M* = 6.20°C, SD = 5.06) and dates only condition combined (*M* = 7.31°C, SD = 5.41), *t*(312) = −3.09, *p* = 0.002. We also performed a 3 × 2 cross‐tabulated Pearson chi‐square test (condition × judgment accuracy), which showed that the proportion of accurate judgments (i.e., 0°C, 1°C, or 2°C) in the dates and temperatures condition (*n* = 53; 52.5%) was significantly higher than in the no labels condition (*n* = 28; 25.7%) and the dates only condition (*n* = 18; 17.1%), *χ* ^2^ = 32.37, df = 2, *p* < 0.001. As in Study 1, a Pearson correlation test identified a strong significant and negative relationship between our sample's mean graph literacy scores (*M* = 2.66, SD = 0.97) and their judgments of the past temperature change, *r* = −0.26, *N* = 315, *p* < 0.001.

**TABLE 4:** Study 2: Participants’ mean responses to questions concerning (i) past and future changes in annual average global temperature, (ii) risk perceptions of climate change, (iii) willingness to adopt mitigation behaviors, (iv) stripe graph evaluations, (v) affective reactions to climate change, and (vi) percentage of funds allocated to societal issues (*N* = 315).

Item	No labels condition mean (SD)	Dates only condition mean (SD)	Dates and temperature condition mean (SD)	Total mean (SD)
***Average annual global temperature changes***				
Average global temperature change since 1850	6.20°C (5.06)	7.31°C (5.41)	4.86°C (4.76)	6.14°C (5.17)
Average global temperature change between the present time and 2050	6.42°C (5.53)	7.20°C (6.07)	5.35°C (5.76)	6.34°C (5.81)
***Risk perceptions***				
*(Response scale: 0 = strongly disagree, 10 = strongly agree)*				
Humanity is not doing enough to tackle climate change	7.47 (2.56)	7.71 (2.41)	7.39 (2.71)	7.52 (2.56)
The average global temperature will rise to dangerous levels during this century	7.15 (2.70)	7.49 (2.48)	7.54 (2.68)	7.39 (2.62)
Climate change poses a threat to the existence of humanity during this century	6.51 (2.97)	6.77 (2.97)	6.41 (3.08)	6.57 (3.00)
The individual actions I can take to tackle climate change are too small to make a difference to global annual temperatures	7.28 (2.73)	7.23 (2.53)	7.39 (2.68)	7.30 (2.64)
Overall perceived risk scale				7.19 (2.32)
***Willingness to employ mitigation behaviors***				
*(Response scale: 0 = not at all, 10 = extremely willing)*				
Reduce annual air travel by 20%	5.86 (3.34)	5.59 (3.36)	5.68 (3.41)	5.71 (3.36)
Reduce annual road travel in personal vehicle by 20%	5.64 (3.36)	5.25 (3.25)	5.32 (3.22)	5.41 (3.27)
Reduce annual household energy consumption by 20%	6.25 (2.89)	5.62 (3.01)	6.01 (2.79)	5.96 (2.90)
Reduce annual consumption of consumer goods and services by 20%	5.91 (2.79)	5.66 (3.00)	5.87 (2.92)	5.81 (2.89)
Vote for political party/candidate committed to reducing or stopping global warming	6.21 (3.23)	6.51 (3.24)	5.79 (3.32)	6.18 (3.26)
Vote for a political party/candidate committed to increasing taxes on fossil fuels	5.59 (3.35)	6.09 (3.48)	5.57 (3.47)	5.75 (3.43)
Talk about climate change with others who do not agree with my view on this topic	5.46 (3.02)	5.58 (2.96)	5.74 (3.10)	5.59 (3.02)
Overall willingness to mitigate scale				5.77 (2.62)
***Stripe graph evaluations***				
Like the way the information in the graph is presented *(Response scale: 0 = do not like it at all, 10 = like it a lot)*	5.20 (2.78)	5.38 (3.02)	5.65 (2.84)	5.41 (2.88)
Trust the information in the graph *(Response scale: 0 = not trust at all, 10 = complete trust)*	5.08 (2.63)	5.70 (2.48)	5.75 (2.27)	5.50 (2.48)
Think the graph provides accurate information *(Response scale: 0 = not at all accurate, 10 = extremely accurate)*	4.77 (2.61)	5.37 (2.54)	5.98 (2.25)	5.36 (2.52)
Graph is helpful for understanding global temperature changes *(Response scale: 0 = not at all helpful, 10 = extremely helpful)*	5.51 (3.00)	5.45 (3.03)	6.59 (2.70)	5.84 (2.95)
Overall graph evaluation scale				5.53 (2.40)
***Affective reactions to global temperature changes***				
*(Response scale: 0 = not at all, 10 = a great deal)*				
Happiness	1.02 (1.59)	1.23 (1.45)	1.55 (2.07)	1.26 (1.72)
Hope	2.45 (2.17)	2.50 (2.41)	2.41 (2.21)	2.45 (2.26)
Inspiration	2.01 (2.24)	2.26 (2.22)	2.26 (2.24)	2.17 (2.23)
Awe	2.39 (2.39)	3.11 (2.68)	2.98 (2.60)	2.82 (2.57)
Curiosity	4.75 (2.54)	5.16 (2.68)	4.85 (2.55)	4.92 (2.59)
Overall positive affect scale				2.72 (1.68)
Guilt	4.83 (3.02)	4.85 (2.91)	4.39 (2.97)	4.70 (2.97)
Sadness	6.61 (2.82)	6.80 (2.73)	6.40 (3.10)	6.61 (2.88)
Fear	6.02 (2.87)	6.36 (2.57)	6.29 (2.99)	6.22 (2.81)
Anger	5.39 (2.99)	5.90 (2.99)	5.73 (3.02)	5.67 (3.00)
Uneasiness	6.85 (2.57)	7.17 (2.43)	6.52 (2.96)	6.85 (2.66)
Overall negative affect scale				6.01 (2.52)
***Percentage of funds allocated to societal issues***				
*(Response scale: 0%–100%)*				
Public health	41.75 (12.54)	40.87 (11.51)	41.54 (12.49)	41.39 (12.16)
Environment and climate change	28.34 (13.53)	27.95 (11.10)	27.96 (14.94)	28.09 (13.22)
Crime and policing	29.91 (13.16)	31.18 (10.70)	30.50 (12.17)	30.52 (12.04)

*Note*: Data is displayed separately for the no labels condition (*n* = 109), dates only condition (*n* = 105), dates and temperatures condition (*n* = 101).

Our sample's mean estimate of the average global temperature change between the present time and 2050 was 6.34°C (SD = 5.81). Although the estimates by participants in the dates and temperatures condition (*M* = 5.35°C, SD = 5.74) were lower than in the no labels (*M* = 6.42°C, SD = 5.53) and dates only conditions (*M* = 7.20°C, SD = 6.07), a one‐way ANOVA identified that the differences in these “future temperature” estimates were just short of statistical significance, *F*(2, 312) = 2.67, *p* = 0.071. However, a planned contrast showed that temperature judgments in the dates and temperatures condition were significantly lower than judgments in the no labels condition and dates only condition combined, *t*(312) = −2.10, *p* = 0.037. Again, a Pearson correlation test identified a strong significant and negative relationship between our sample's graph literacy scores and their estimates of the future temperature change, *r* = −0.33, *N* = 315, *p* < 0.001.

Table 4 shows the mean responses to the main measures in the questionnaire, which the participants completed while being able to view the condition‐relevant graph. We created separate scales from the items used to measure, respectively, perceived risk (Cronbach's *α* = 0.88), graph evaluations (Cronbach's *α* = 0.91), positive affective (Cronbach's *α* = 0.79), negative affect (Cronbach's *α* = 0.93), and willingness to engage in mitigation behaviors (Cronbach's *α* = 0.92). We then performed separate one‐way ANOVAs to assess for between‐conditions differences in the mean scores on each of these scales. The results identified a significant difference for overall graph evaluations, *F*(2, 312) = 3.41, *p* = 0.034, but not for the other four constructs, *F*s(2, 312) ≤ 1.21, *p*s ≥ 0.299. Bonferroni post hoc analysis revealed (*p* = 0.030) that the observed difference in graph evaluations was attributable to higher graph evaluations in the dates and temperatures condition (*M* = 6.00, SD = 2.15) compared to graph evaluations in the no labels condition (*M* = 5.14, SD = 2.47).

Similar to the results observed in Study 1, a further ANOVA identified no difference in the participants’ mean allocation of UK Government funds to “environment and climate change” in the no labels condition (*M* = 28.34%, SD = 13.53), the dates only condition (*M* = 27.95%, SD = 11.10), and the dates and temperatures condition (*M* = 27.96%, SD = 14.94), *F*(2, 312) = 0.03, *p* = 0.971 (see Table 4).

We performed an exploratory multiple regression analysis to determine which factors might be associated with our participants’ willingness to engage in climate change mitigation actions. We performed a forced entry linear regression with willingness to engage in mitigation behaviors as the outcome variable and overall perceived risk, condition (0 = “no labels” and “dates only” conditions, 1 = “dates and temperatures” condition), past temperature change, future temperature change, positive affect, negative affect, graph literacy, worldviews, eco‐anxiety, and prior experience of stripe graphs as the predictors. Tables 5 and 6 show the correlations and coefficients, respectively. The analysis revealed that perceived risk, past temperature change, negative affect, worldviews, and eco‐anxiety were significant predictors, with the regression model explaining 62% of the variance in willingness to engage in mitigation behaviors.^2^

**TABLE 5:** Study 2: Correlations between assessed variables (*N* = 315).

	1	2	3	4	5	6	7	8	9	10	11
1. Overall mitigation willingness	1										
2. Overall perceived risk	0.712^***^	1									
3. Condition	−0.016	−0.004	1								
4. Past temperature	0.126^*^	0.067	−0.171^***^	1							
5. Future temperature	0.132^**^	0.133^**^	−0.117^*^	0.680^***^	1						
6. Positive affect	0.159^**^	0.104^*^	0.035	0.051	0.119^*^	1					
7. Negative affect	0.736^***^	0.792^***^	−0.039	0.060	0.148^**^	0.179^***^	1				
8. Graph literacy	−0.040	0.022	0.002	−0.262^***^	−0.329^***^	−0.114^*^	−0.031	1			
9. Worldviews	0.490^***^	0.496^***^	−0.087	−0.035	−0.042	−0.056	0.449^***^	0.029	1		
10. Eco‐anxiety	0.348^***^	0.328^***^	−0.013	0.084	0.228^***^	0.204^***^	0.370^***^	−0.053	0.097^*^	1	
11. Stripe graph past experience	0.110^*^	0.070	0.166^**^	−0.139^**^	−0.081	0.031	0.060	0.058	0.128^*^	0.112^*^	1

* *p* < 0.05.

** *p* < 0.01.

*** *p* < 0.001.

**TABLE 6:** Study 2: Regression of assessed variables on overall willingness to engage in climate change mitigation behaviors (*N* = 315).

	Unstandardized coefficients		Standardized coefficients
	*b*	SE	*β*
Overall perceived risk	0.311	0.068	0.275^***^
Condition	0.121	0.205	0.022
Past temperature	0.068	0.025	0.134^**^
Future temperature	−0.033	0.023	−0.074
Positive affect	0.075	0.057	0.048
Negative affect	0.422	0.062	0.405^***^
Graph literacy	−0.055	0.102	−0.020
Worldviews	0.240	0.061	0.165^***^
Eco‐anxiety	0.469	0.226	0.082^*^
Stripe graph past experience	0.043	0.035	0.045
*R^2^*			0.624^***^
*F*(10,304)			50.40

*Note*: Variance inflation factor (VIF) and tolerance statistics showed no evidence of multicollinearity.

* *p* < 0.05.

** *p* < 0.01.

*** *p* < 0.001.

#### 2.2.5 Discussion

The results of Study 2 suggest that exposure to a stripe graph featuring date and temperature labels (cf., featuring no labels) can help audiences acquire more accurate knowledge of past global temperature changes. Similarly, the results indicate that exposure to a stripe graph featuring date and temperature labels (cf., featuring no labels or date labels only) may help audiences make estimates of future temperature changes that align more closely with projections from current climate models. Nonetheless, it is worth noting that our participants’ knowledge of past temperature changes (and estimates of future changes) still tended to be overestimations. This indicates that there is scope to further improve the design of stripe graphs featuring date and temperature labels so that this format can better help audiences extract more accurate information.

The popularity of the “climate warming” stripe graph has been attributed, in part, to the absence of technical features such as labeled axes (Hawkins 2019; Rosch 2023). Indeed, the unlabeled stripe graph was “*designed to be as a simple as possible*” and to “*enable communication with minimal scientific knowledge required to understand their meaning*” (www.showyourstripes.info/faq). However, consistent with Grøndahl et al. (2011), Study 2 found that the participants had a significantly greater preference for the labeled graph than for the unlabeled graph. This suggests that communicators should be more willing to use stripe graphs with labels and, in doing so, might elicit greater engagement with their target audiences and/or help them obtain more accurate knowledge. However, it should be noted that the participants in our study were specifically required to provide numerical temperature estimates, and, therefore, they may have had a task‐related incentive to favor a labeled (cf., unlabeled) graph in which more explicit and precise data on temperature and time periods were available.

Several findings from Study 2 bore strong similarities to those observed in Study 1. For example, Study 2 found that lower graph literates were more prone to providing inaccurately high estimates of past and future temperature changes. Study 2 also found that exposure to different stripe graphs did not lead to significant variations in risk perceptions and affect. Furthermore, consistent with Study 1, the Study 2 regression model found that perceived risk, negative affect, worldviews, and eco‐anxiety were all significant predictors of willingness to engage in mitigation behaviors, thus reaffirming the strong role that each of these variables plays in motivating environmental actions. However, in contrast to the results in Study 1, the Study 2 regression model identified past temperature estimates as a predictor of willingness to engage in mitigation behaviors. Indeed, the analysis revealed a positive correlation between estimates of past temperatures and mitigation willingness (see Table 5). On one hand, this finding is not surprising because one might expect higher past temperature changes (i.e., greater climate change) to motivate the desire for remedial actions. On the other hand, it raises the ethical dilemma for communicators about whether they should help audiences develop more accurate (i.e., lower) knowledge of past (estimates of future) temperature changes if this depresses the audiences’ willingness to engage in mitigation behaviors. We suggest that the answer is to engage in communication strategies that both elicit accurate knowledge/estimates but that also instill a clear understanding of the consequences of such temperature changes.

## 3 General Discussion

We conducted two studies that, to the best of our knowledge, are the first to empirically assess the relative influence of climate stripe graphs on knowledge, perceptions, and behavioral intentions among a lay audience. The results of Study 1 found no evidence that unlabeled climate stripes graphs improve knowledge of past global temperature changes or the accuracy of estimates of future temperature changes. The study also indicated that the graphs had little influence on risk perceptions, affect, or environmental decision‐making. Notably, Study 1 did indicate that viewing a stripe graph was associated with a lower willingness to engage in mitigation behaviors and that seeing a stripe graph for the first time led to an increase in past and future estimates, thus making those estimates less accurate. Despite these potential drawbacks of the stripe graphs, the findings also showed that the blue–red unlabeled graph was, in relative terms, perceived to be likeable, trustworthy, accurate, and helpful. Our second study identified that a climate stripes graph featuring date and temperature labels (cf., no labels) helped individuals to acquire more accurate knowledge of past global temperature changes and make more accurate estimates of future temperature changes. However, the temperature judgments were still higher than those reported in scientific data and models. Again, the results of Study 2 indicated that the climate stripe graphs did not lead to significant variations in risk perceptions, affect, or environmental decision‐making.

The design of Hawkins’ “climate warming” stripe graph has proved to be extremely popular among science/risk communicators and lay audiences and has undeniably demonstrated a remarkable capacity to achieve its intended aims of being “… *as simple as possible, and to start conversations about our warming world and the risks of climate change*” (#ShowYourStripes 2025). In the global effort to tackle climate change, there are obvious benefits to capitalizing on the popularity of the stripe graph as a means of engaging people with information on environmental issues and, hopefully, motivating them to adopt, demand, and support mitigation actions. Nonetheless, it will be important that those who use stripe graphs have a clear understanding of the format's strengths and limitations and of what influence the format may have on the target audience, including influences that may be undesirable. The present studies take the first steps towards providing such an understanding.

Communicators of environmental data who wish to improve the audience's scientific literacy or affect their risk perceptions and behaviors will need to consider the extent to which unlabeled stripe graphs can help to achieve these aims. Communicators should be mindful that, despite their immense popularity among public audiences, unlabeled stripe graphs may have little direct influence on knowledge, risk perceptions, and behavioral intentions. Indeed, our results suggest that audiences prefer stripe graphs with labeled axes and that this design is more effective at enhancing accurate knowledge. Hence, risk/science communicators should be more willing to disseminate labeled stripe graphs. Moreover, communicators might even consider simultaneously using both the labeled and unlabeled formats so that the former enhances engagement while the latter enhances knowledge transfer.

It is also worth noting that because unlabeled stripe graphs feature bars/stripes of equal heights, it is the stripe colors and saturations that are the audience's sole means of differentiation between data values. This design differs from the labeled stripe graph that we presented in Study 2, which uses variations in the bar/stripe heights to represent temperature variations. The variation in the bar/stripe heights makes the graph more consistent with the more commonly encountered bar graph and, therefore, may be another reason why audiences may find this design more helpful and likeable.

### 3.1 Limitations and Future Research Directions

The increasing popularity and use of stripe graphs contrasts sharply with the lack of empirical knowledge about the efficacy of this graphical approach for achieving communication objectives. Although our two studies provide some preliminary insights, there remains extensive scope for future research concerning this graphical format. First, we recommend that researchers compare stripe graphs to other graphical risk communication formats (e.g., icon arrays, line graphs, and temperature maps), with a view to identifying the relative strengths of this format. Indeed, graphical risk communications can vary greatly in their design and complexity and, consequently, can elicit significant variations in cognitive, affective, and behavioral responses among audiences (Kurz‐Milcke et al. 2008; Rogers 2025; Schuster et al. 2024). Understanding how stripe graphs compare to and can complement other formats (and recognizing when it might be better not to use stripe graphs) can prove extremely valuable in effectively achieving science/risk communication objectives. Second, our studies only assessed stripe graphs that communicate climate change data. Hence, it would be useful to assess the efficacy of stripe graphs for communicating other environmental risks and other non‐environmental risks (e.g., health risks and financial risks). Third, although we assessed the relative influence of two different color combinations (i.e., blue–red vs. yellow–purple), it is possible that other color combinations may elicit different responses from audiences, and this should be assessed. Furthermore, responses might vary because of how the audiences’ cultural backgrounds influence their semantic interpretation of the graphs’ colors and layout (Börner et al. 2019), and thus, these factors could also be assessed. Fourth, our second study identified that the participants’ judgments of the past annual average global temperatures were positively related to their willingness to engage in mitigation actions. However, this relationship was not evident in our first study. The difference in findings leads to some uncertainty about the strength and nature of this potential relationship, which should be explored in future research. Fifth, the issue of climate change is often associated with political polarization and partisanship (Cole et al. 2023; Falkenberg et al. 2022), and, therefore, climate stripe graphs could be championed or castigated by political factions to support their political agendas. Hence, we suggest that future studies could examine the extent to which the direction and strength of an individual's political affiliations might influence the ways in which they encounter, interpret, and use stripe graphs. Finally, our participants viewed stripe graphs with little or no supporting information. There is a growing body of literature that indicates the efficacy of data visualizations can be substantially enhanced by integrating such visualizations with instructional guidance and supporting narratives (known as “Data Storytelling [DS]”) (Shao et al. 2024). Hence, future studies could explore the role of DS in enhancing the capacity of stripe graphs to achieve specific communication objectives. It is via further research of stripe graphs that a comprehensive understanding of the format's impacts and efficacy can be developed and, therefore, that the format's design, use, and immense popularity can be exploited to effectively enhance knowledge, risk literacy, and motivate mitigation actions.

## Conflicts of Interest

The authors declare no conflicts of interest.

## Endnotes

1 To further explore the predictors of mitigation willingness, we re‐ran our multiple regressions for Study 1, first with participants who provided accurate future temperature predictions (1°–4°; *n* = 151) and then with those who provided inaccurate predictions (all other values; *n* = 164). For the “accurate” participants, condition (viewing vs. not viewing a stripe graph) was not a significant predictor of willingness to engage in mitigation behaviors. For the “inaccurate” participants, condition was a significant predictor: as in the main Study 1 regression, it was those that saw a stripe graph who were less willing to engage in mitigation behaviors. *G*Power* analysis indicated that both regressions were underpowered to detect even medium effect sizes. Thus, these results should be interpreted with considerable caution.

2 To further explore the predictors of mitigation willingness, we re‐ran our regression analyses for Study 2, first using participants with accurate predictions (1°–4°; *n* = 157) and then using those with inaccurate predictions (all other values; *n* = 158). For the “accurate” participants, condition significantly predicted willingness to engage in mitigation behaviors. For the “inaccurate” participants, consistent with the main Study 2 regression results, condition did not predict willingness to engage. *G*Power* analysis indicated that both regressions were underpowered to detect even medium effect sizes. Thus, these results should be interpreted with considerable caution.
